# Prosthetic Mitral Valve Obstruction with Left Atrial Appendage Thrombus: A Therapeutic Dilemma

**DOI:** 10.7759/cureus.5011

**Published:** 2019-06-26

**Authors:** Venkatesh Ravi, Aswathi Chandran, Ralph Matar, Priyanjali Pulipati, Neha Yadav

**Affiliations:** 1 Cardiology, John H Stroger, Jr. Hospital of Cook County, Chicago, USA; 2 Gastroenterology, Hepatology and Nutrition, University of Texas Health Science Center at Houston, Houston, USA; 3 Medicine, Jawaharlal Nehru Medical College, KLE University, Belgaum, IND

**Keywords:** prosthetic mitral valve obstruction, pannus, thrombus, thrombolytics

## Abstract

Valvular obstruction is a rare but life-threatening complication of mechanical prosthetic valves that raises significant challenges in management. We describe a unique case of mechanical mitral valve obstruction with co-existing left atrial appendage (LAA) thrombus. A 48-year-old man with a past medical history of atrial fibrillation and mechanical mitral valve replacement 18 months prior, presented with symptoms of new onset heart failure for 10 days. INR on presentation was sub-therapeutic. Trans-thoracic and trans-esophageal echocardiography revealed prosthetic mitral valve obstruction with mobile, echogenic masses seen on the mechanical valve as well as LAA, suggestive of thrombus. His clinical course rapidly deteriorated and he developed cardiogenic shock. He was deemed to have prohibitive risk for emergent surgical intervention. He received trial of thrombolytic therapy, with partial improvement of hemodynamic parameters and a mild decrease in thrombus burden. He then underwent surgical intervention with a favorable outcome. Intra-operative visualization of the prosthetic valve revealed a combination of pannus and thrombus. Prosthetic valve function should be promptly assessed in patients presenting with heart failure symptoms, as clinical deterioration can be rapid. Acute presentation, history of inadequate anticoagulation and appearance of soft mass on an echocardiogram, are suggestive of thrombus as the etiology of valve obstruction. However, thrombus and pannus are known to frequently co-exist. Emergent surgery is the recommended management strategy in patients with left-sided prosthetic valve thrombosis with the New York Heart Association (NYHA) III or IV symptoms, due to a lower rate of thrombo-embolism, major bleeding, and recurrent prosthetic valve thrombosis when compared with thrombolytic therapy. Slow-infusion, low-dose thrombolytics were recently shown to have favorable outcomes and can be considered when surgery is not available or the patient is deemed to have prohibitive surgical risk.

## Introduction

Valvular obstruction in mechanical prosthetic valves is usually caused by thrombus formation, pannus ingrowth, a combination of both or rarely a vegetation. Thrombus formation depends on anticoagulation status, valve position, presence of atrial fibrillation, and/or ventricular dysfunction. Pannus refers to an overgrowth of fibrous tissue due to an inflammatory reaction, usually after many years of valve replacement and may have an overlying thrombus layer. In a study of 100 patients with mechanical valve obstruction who underwent surgical intervention, predominant etiologies were thrombus, pannus, and a combination of both in 77.7%, 10.7%, and 11.6% of the cases respectively [1]. However, pannus formation of varying degrees of severity was seen in about 46% of these patients, and represents an important reason for the failure of thrombolysis [1]. Establishing the etiology of obstruction is challenging, but has a significant impact on the management, as thrombolytic regimens have more likelihood of success in patients with thrombus. Presence of a left atrial appendage (LAA) thrombus in addition to left-sided prosthetic valve thrombotic obstruction raises an additional therapeutic dilemma, due to high risk of embolic complications from thrombolytic therapy [2]. In this report, we describe a unique case of mechanical mitral valve obstruction and co-existing LAA thrombus, in which systemic thrombolytic therapy was attempted, prior to definitive surgical management.

## Case presentation

A 48-year-old man presented to our hospital with new onset shortness of breath, orthopnea, and lower extremity edema of 10 days duration. Past medical history was significant for diabetes and atrial fibrillation. He also had a history of rheumatic heart disease with severe mitral stenosis and moderate mitral regurgitation for which he underwent mitral valve replacement with a #29 mm St Jude’s mechanical valve, 18 months prior to presentation. His vitals on presentation were: heart rate of 79 beats/min, blood pressure of 119/69 mmHg, temperature of 98.9^o^F, and respiratory rate of 14 breaths/min. His oxygen saturation was 96% on room air. Examination showed elevated jugular venous distension, bilateral pedal edema, irregular heart rate, and metallic S1 click with no clear evidence of murmur. Chest auscultation revealed diffuse rhonchi and expiratory wheezing. Chest X-ray and CT of the chest showed pulmonary edema and bilateral pleural effusions. His INR on admission was 1.67. On further review, his INR was sub-therapeutic on multiple instances since his surgery. Initial ECG revealed atrial fibrillation with a rate-controlled ventricular response. He was started on intravenous diuresis and enoxaparin for bridging anticoagulation.

**Figure 1:**
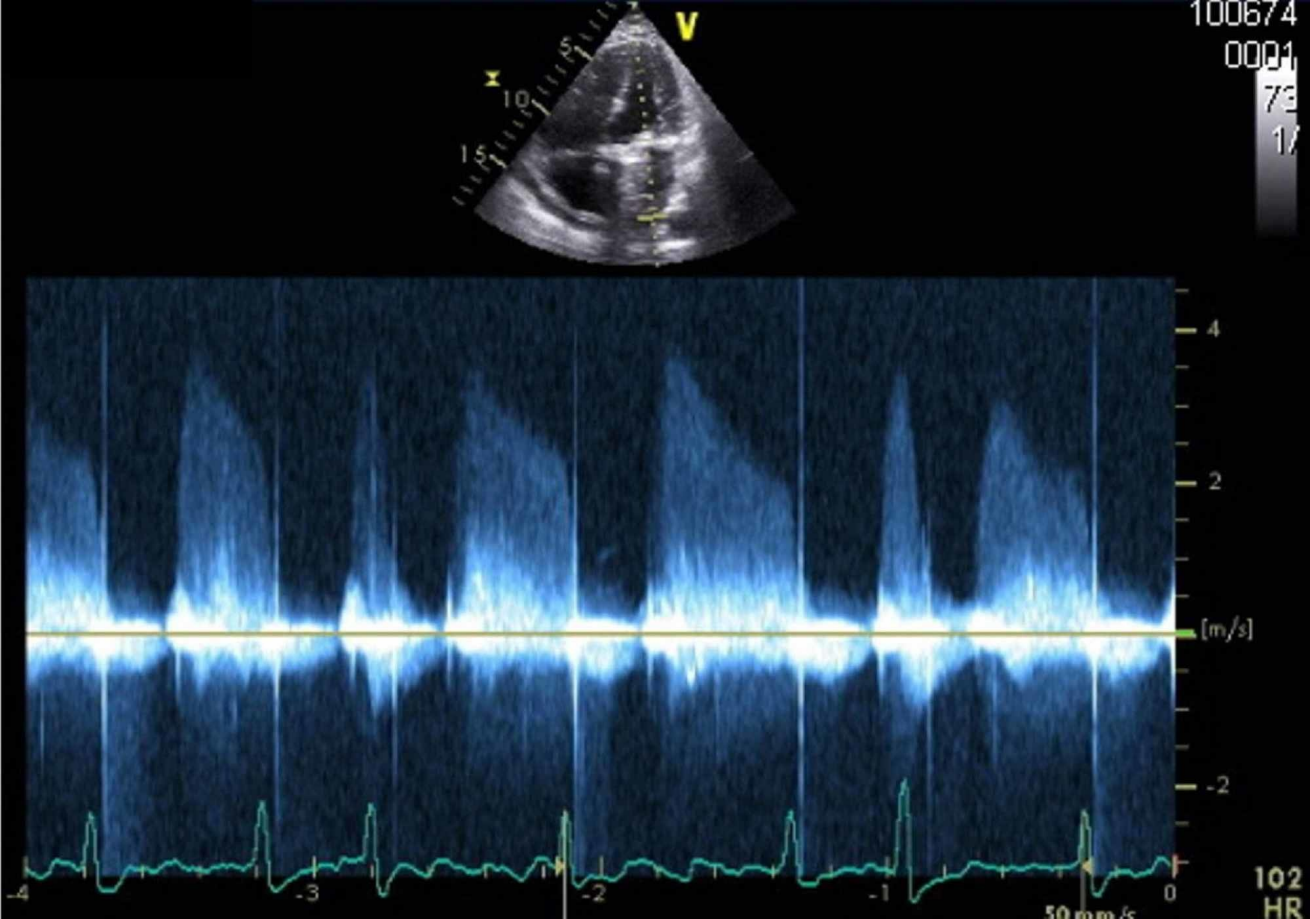
Transthoracic echocardiography, apical four chamber view. Continuous doppler across the mitral valve, demonstrating elevated trans-mitral gradient of 23 mmHg (averaged over five beats) at a heart rate of 102 beats/min.

**Figure 2:**
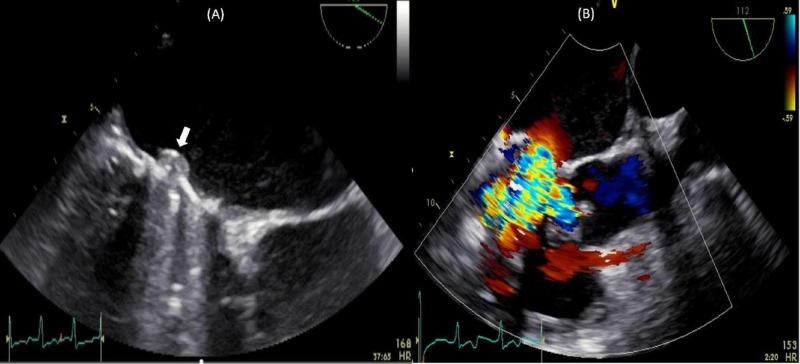
Trans-esophageal echocardiography mitral valve focused view. Demonstrating a mass (white arrow) on the mechanical mitral valve concerning for thrombus (A) and turbulent flow with color doppler across the mitral valve (B).

**Figure 3:**
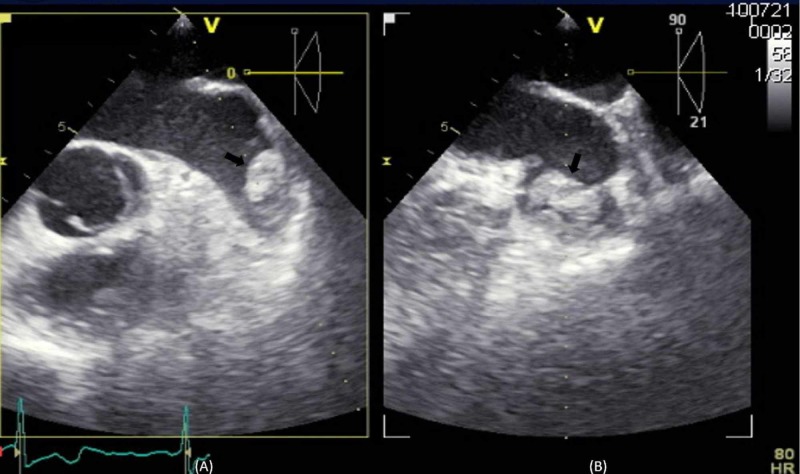
Trans-esophageal echocardiogram biplane view of the left atrial appendage. Demonstrating the mass (black arrow) concerning for thrombus in transverse (A) and longitudinal axis (B) in the left atrial appendage.

**Figure 4:**
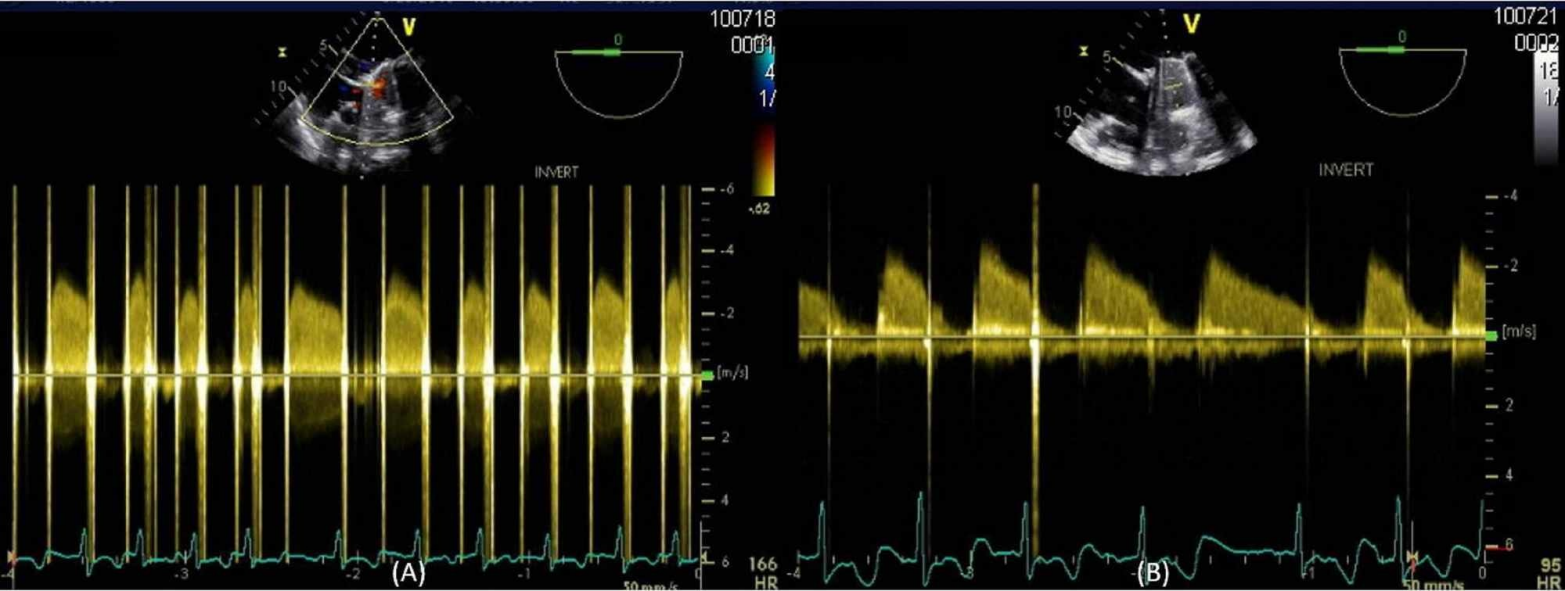
Trans-esophageal echocardiogram with continuous wave doppler across the mitral valve. Demonstrating elevated mean trans-mitral gradient of 24 mmHg (averaged over five beats) at heart rate of 166 beats/min before initiation of thrombolytics (A) and a trans-mitral gradient of 15 mmHg (averaged over five beats) at a heart rate of 95 beats/minute after trial of thrombolytics (B).

His clinical condition deteriorated over the course of 24 h and he developed severe respiratory distress. He was tachycardic with a heart rate of 140 beats/min and hypotensive with a blood pressure of 84/52 mmHg. He was admitted to cardiac intensive care unit with a diagnosis of cardiogenic shock, initiated on mechanical ventilation and pressor support with phenylehprine. Transthoracic echocardiography (TTE) revealed normal left ventricular ejection fraction and size. Mitral valve leaflet motion was reduced with an increased trans-valvular velocity of 3.2 m/s. Mean trans-mitral gradient was elevated to 23 mmHg at a heart rate of 102 beats/min (Figure 1). Trans-esophageal echocardiography (TEE) confirmed mechanical mitral valve stenosis with a fixed leaflet. A medium-sized, spherical, echogenic, mobile mass, measuring at least 0.8 cm^2^ was visualized on the atrial aspect of the mitral valve, suggestive of a thrombus (Figure 2A-B). There was also a large, mobile, echogenic mass in the LAA, consistent with thrombus (Figure 3A-B). The left atrium was dilated with severe spontaneous contrast (‘smoke’) in the cavity. Cardiovascular surgery deemed the patient to have prohibitive risk for emergent surgical intervention and recommended a trial of thrombolytics. Intravenous alteplase was administered as a 10-mg bolus followed by 90-mg infusion over two hours. Therapeutic anticoagulation with heparin was initiated after the completion of alteplase infusion. His tachycardia and pressor requirement improved immediately following the use of thrombolytics. Post-thrombolytic TTE showed a peak velocity of 2.5 m/s and a mean gradient of 15 mmHg at a heart rate of 98 beats/min while post-thrombolytic TEE re-demonstrated a fixed leaflet (Figure 4A-B). The mobile, echogenic mass on the valve appeared to be slightly reduced and measured 0.6 cm2. The LAA mass also appeared slightly smaller in size but had increased mobility. His neurologic examination remained stable and was not suggestive of clinically significant embolization.

**Figure 5:**
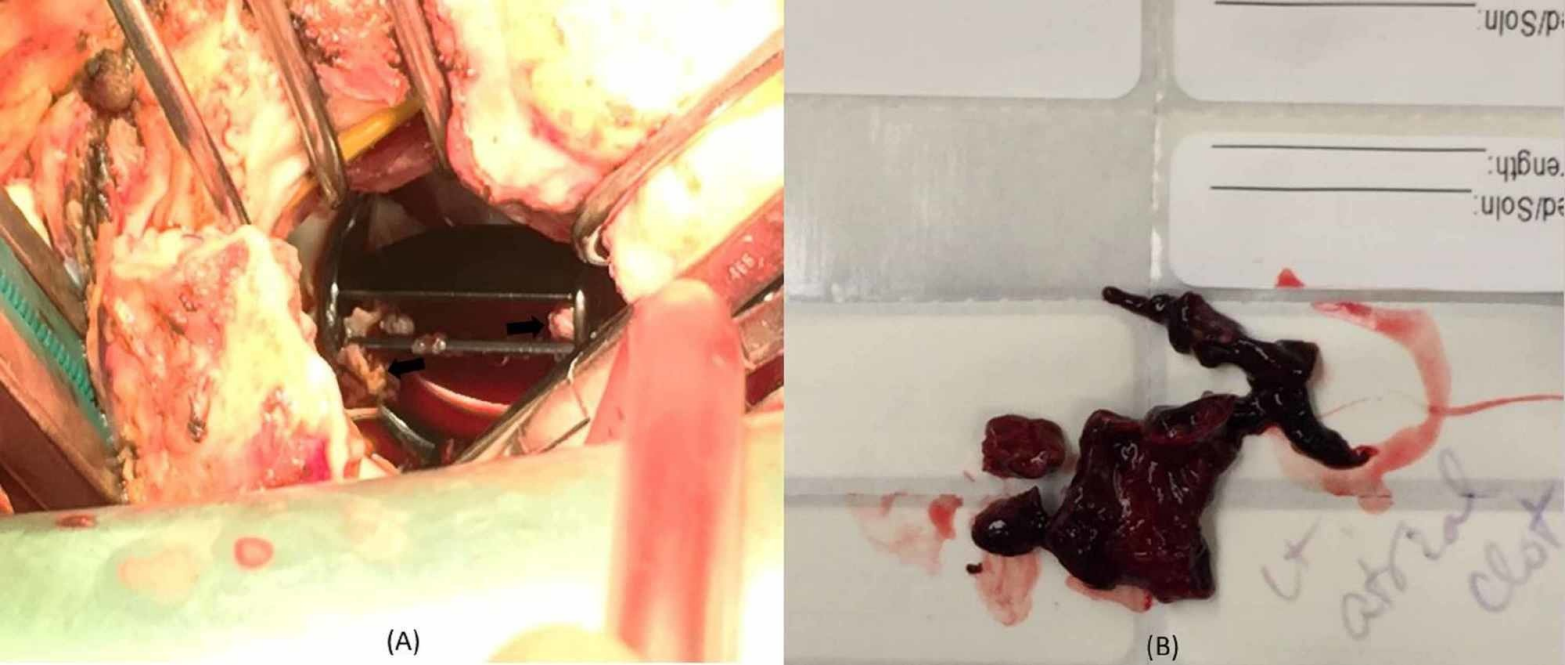
Perioperative images. Demonstrating the pannus overgrowth (arrows) restricting the mechanical mitral valve leaflets (A) and left atrial appendage thrombus after removal (B).

Repeat administration of thrombolytics was not performed given LAA thrombus hypermobility. Following extensive discussion with family, shared multi-disciplinary decision was made to proceed with surgical intervention. He underwent surgical mitral valve replacement with #31mm Mosaic bio-prosthetic valve, extraction of left atrial thrombus, and exclusion of LAA by ligation. Intra-operative findings showed the explanted mechanical mitral valve covered with pannus and thrombus at the medial corner of the valve and at the hinged area obstructing one of the leaflets. There was also pannus encasing the circumference (Figure 5A). A large thrombus was successfully extracted from the LAA (Figure 5B). Pathology of the specimen was consistent with fibro-collagenous tissue with focal mild chronic inflammation, focal calcification, focal degenerative changes, and thrombus. Post-operative TEE showed normal functioning of the newly implanted bio-prosthetic mitral valve. He did not have any evidence of thrombo-embolic complications during hospitalization and continues to follow up in clinic one year later with good functional status.

## Discussion

Our patient was hemodynamically unstable with no urgent surgical intervention available as he was deemed to have prohibitive risk. He was also a poor candidate for thrombolytic therapy given the New York Heart association (NYHA) IV symptoms, large thrombus burden including LAA thrombus. A trial of thrombolytics was offered as salvage. He had a partial improvement in hemodynamic parameters and a slight decrease in thrombus burden prior to successful surgical intervention.

The prevalence of mechanical prosthetic valve thrombosis is 0.3%-1.3% per patient-year in developed countries and as high as 6.1% in developing countries [3]. The risk of thrombosis is increased in patients with mitral valve prosthesis, atrial fibrillation, previous thromboembolic events, and hypercoagulable state [4]. Inadequate anticoagulation and acute presentation of symptoms are usually suggestive of thrombus as etiology of prosthetic valve obstruction. However, frequently both thrombus and pannus seem to coexist as seen in our case [1]. The usual period for tissue overgrowth and pannus formation in a mechanical prosthesis is six months or longer after implantation, during which an ingrowth of peri-annular tissue would gradually immobilize the moving elements of the prosthesis [1, 5]. There are some case reports where a shorter interval has been reported [6]. A study of 24 patients with mechanical valve obstruction that evaluated clinical and echocardiographic findings to differentiate thrombus from pannus, demonstrated inadequate anticoagulation to be the best objective clinical parameter for prediction of thrombus, with a sensitivity of 79% and a specificity of 90% [5]. Among the TEE parameters, the appearance of soft mass defined as having ultrasound echo density similar to the myocardium was predictive of thrombus, with a sensitivity of 86% and specificity of 80% [5]. The presence of either inadequate anticoagulation or a soft mass by TEE improved the predictive power of either parameter alone, with a sensitivity of 93% and specificity of 80% [5]. Thrombi were also noted to be larger than pannus, mostly due to the extension of thrombi into the left atrium in prosthetic mitral valves [1].

Management options for symptomatic left-sided thrombotic obstruction include thrombolytic therapy and surgery. According to the 2014 valvular heart disease guidelines, the only Class 1 recommendation for patients with left-sided prosthetic valve thrombus and NYHA III or IV symptoms was an emergency surgery. This was based on evidence that surgery when compared with thrombolytic therapy, was associated with a lower rate of thromboembolism (1.6% vs 16%), major bleeding (1.4% vs 5%), and recurrent prosthetic valve thrombosis (7.1% vs 25.4%) [7-8]. Emergency surgery is also recommended for patients with left-sided prosthetic valve thrombus with a mobile or large thrombus (>0.8 cm^2^) [1, 9-10]. Those with large thrombus burden (>0.8 cm^2^) had a 2.4-fold rate of complications per 1.0 cm^2^ increase in size, making surgery the optimal intervention in that case [10]. However, it has to be highlighted that there were no randomized trials directly comparing surgery with thrombolytics and the previously mentioned rates are from meta-analysis of several individual trials that used either surgery or thrombolytics in different patient populations.

The management of patients is more challenging when they satisfy the above criteria for emergent surgery but are deemed a high-risk surgical candidate or surgery is not immediately available. The recent update to valvular heart disease guidelines provided a Class 1 recommendation for the option of urgent use of slow-infusion, low-dose thrombolytics along with the option for surgical intervention [8-9, 11-13]. This change in recommendation was based on recent reports showing >90% success rates with <2% embolic and major bleeding rates, using a trans-esophageal echocardiogram guided protocol, with slow infusion over 25 h of low dose alteplase at 25 mg with an option of repeat thrombolytics for the failure of therapy [14]. However, the success rates, defined as >75% reduction in thrombus area and/or length, were lower and complications were higher in patients with NYHA IV symptoms, atrial fibrillation, and large thrombus area [14]. The NYHA class IV status was an independent predictor of an unsuccessful outcome with an odds ratio of 14.3; 95% CI, 1.8-111.1; p = 0.012 [14]. Notably, patients with LAA thrombus were excluded from the trial. The decision for emergency surgery vs thrombolytic therapy was recommended to be made based on multiple factors, including the availability of surgical expertise and the clinical experience with both treatments.

There have been isolated reports of successful systemic thrombolysis of LAA thrombus, but the safety is not well established [15-16]. A strategy of routine thrombolytic therapy followed by surgery might delay initiation of surgical therapy if it becomes necessary and increase the risk of complications, hence is not routinely recommended [17]. Left atrial catheter directed administration of thrombolytics in patients with contraindication to systemic thrombolytics has also been successfully described in isolated case reports but requires more extensive evaluation to determine rates of success and complications [18].

In general, factors that favor thrombolytics included lack of surgical expertise, high surgical risk, NYHA I-III, thrombus size < 0.8 cm^2^, and absence of LAA thrombus [3, 11-12]. Whereas, factors that identify patients at risk for adverse outcomes of thrombolytic therapy include active internal bleeding, history of hemorrhagic stroke, large thrombi, mobile thrombi, systemic hypertension (>200/120 mmHg), hypotension or shock, and NYHA Class III to IV symptoms [3, 11-12].

## Conclusions

Prompt assessment of prosthetic valve function in a patient presenting with heart failure symptoms is of foremost importance, as clinical deterioration can be rapid. Patients with NYHA Class III or IV and evidence of valve obstruction should undergo emergent valve replacement surgery. When emergent surgery is unavailable or deemed to have prohibitive risk, thrombolytic therapy can be considered. The use of thrombolytics vs surgery is a complex decision that requires interdisciplinary collaboration, surgical expertise as well as customization based on patient characteristics. With recent advancements in valve technology and architecture, the life-threatening complications are expected to become less frequent in future. 
